# Dementia caregiving: Predictors of depression, anxiety and sleep duration

**DOI:** 10.1002/alz70858_105054

**Published:** 2025-12-26

**Authors:** Terry R Barclay, Aleta L Svitak, Sally K Gustafson, Clarissa M Howe, Samantha J Sherman, Pamala A Pawloski, Leah R Hanson

**Affiliations:** ^1^ HealthPartners Institute, Minneapolis, MN, USA; ^2^ HealthPartners Center for Memory & Aging, St. Paul, MN, USA

## Abstract

**Background:**

The Minnesota Memory Project is a longitudinal, observational study investigating health, lifestyle and cognitive factors associated with advancing age. The study enrolled several cohorts, including adults providing care for a person with mild cognitive impairment (MCI) or dementia. Prior research has established that the demands of caregiving often lead to diminished self‐care and increased risk of mood disorders, sleep disturbance and a wide variety of health problems. Identifying modifiable factors associated with these poor outcomes can help target interventions to better support caregivers and improve their health and well‐being.

**Method:**

The current analysis examined caregiver participants who completed baseline measurements. Annual research visits included detailed questionnaires and self‐report inventories regarding health history, lifestyle characteristics, caregiving characteristics, mood and other factors. Depression (Center for Epidemiologic Studies Depression Scale), anxiety (State Trait Anxiety Inventory) and sleep (Optimal Sleep Scale) were selected as outcome variables. Regression analyses were used to model the relationship between study outcomes and stress, burden, resilience, coping, social support and exercise frequency.

**Result:**

Caregivers (*N* = 84) had a mean age of 64.6 years and were generally female (78.6%), non‐Hispanic white (97.6%), with a four‐year college degree or greater (65.5%). Most were caring for a spouse (52.4%) or parent (33.3%) living at home (54.5%). Analyses were controlled for caregiver sex, employment status, hours spent providing care, use of paid care/resources, caregiver physical health, history of psychiatric and neurological conditions as well as the care setting of the care receiver. After controlling for all covariates, higher levels of stress and burden and lower levels of resilience and social support were strongly associated with increased symptoms of depression and anxiety. Coping and exercise frequency were inconsistently associated with mood. Lower stress and higher levels of resilience and positive coping were significantly associated with optimal sleep.

**Conclusion:**

More interventions are needed to effectively reduce symptoms of mood and sleep disturbance and to improve overall well‐being among individuals caring for a person with MCI/dementia. Successful strategies will include interventions targeting stress management, burden reduction, expansion of social support and building resilience.

